# Protuberant Electrode Structures for Subretinal Electrical Stimulation: Modeling, Fabrication and *in vivo* Evaluation

**DOI:** 10.3389/fnins.2019.00885

**Published:** 2019-08-27

**Authors:** Pedro González Losada, Lionel Rousseau, Marjorie Grzeskowiak, Manon Valet, Diep Nguyen, Julie Dégardin, Elisabeth Dubus, Serge Picaud, Gaelle Lissorgues

**Affiliations:** ^1^Laboratory ESYCOM, University Paris Est-ESIEE-MLV, Noisy-le-Grand, France; ^2^INSERM, CNRS, Institut de la Vision, Sorbonne Université, Paris, France

**Keywords:** retinal prostheses, microfabrication, 3D microelectrode, FEM, subretinal, electrical stimulation

## Abstract

Many neural interfaces used for therapeutic applications are based on extracellular electrical stimulation to control cell polarization and thus functional activity. Amongst them, retinal implants have been designed to restore visual perception in blind patients affected by photoreceptor degeneration diseases, such as age-related macular degeneration (AMD) or retinitis pigmentosa (RP). While designing such a neural interface, several aspects must be taken into account, like the stimulation efficiency related to the current distribution within the tissue, the bio-interface optimization to improve resolution and tissue integration, and the material biocompatibility associated with long-term aging. In this study, we investigate the use of original microelectrode geometries for subretinal stimulation. The proposed structures combine the use of 3D wells with protuberant mushroom shaped electrode structures in the bottom, implemented on a flexible substrate that allows the *in vivo* implantation of the devices. These 3D microelectrode structures were first modeled using finite element analysis. Then, a specific microfabrication process compatible with flexible implants was developed to create the 3D microelectrode structures. These structures were tested *in vivo* to check the adaptation of the retinal tissue to them. Finally, preliminary *in vivo* stimulation experiments were performed.

## Introduction

Many neural interfaces used for therapeutic applications are based on extracellular electrical stimulation to control cell polarization and thus functional activity. Common examples range from deep brain stimulators for Parkinson’s disease to sensory prostheses such as cochlear implants (Deuschl et al., 2006; Schwalb and Hamani, 2008; Eshraghi et al., 2012). More recently, retinal implants have been designed to restore some visual perception in blind patients affected by photoreceptor degeneration diseases, such as age-related macular degeneration (AMD) and retinitis pigmentosa (RP). Depending on the chosen surgery, the implant may be placed at different locations to intervene at different levels in the visual system: (i) into the subretinal space where the electrode array is located between the inner nuclear layer and the retinal pigment epithelium to replace the lost photoreceptors like Alpha-AMS (Edwards et al., 2018) and Pixium PRIMA^®^ (Hornig et al., 2017) devices, (ii) on the epiretinal position close to the ganglion cell layer as the Argus-II prosthesis (Luo and da Cruz, 2016) *or the Pixium IRIS*^®^. In the case of subretinal implantation, the electrical stimulation is expected to activate the surviving retinal bipolar cells, which transfers the artificial visual information coded as spike signals to the ganglion cells and the optic nerve whereas epiretinal implants aim at direct activation of retinal ganglion cells.

While designing such a neural interface, several aspects must be taken into account: (i) the stimulation efficiency related to the current distribution within the tissue, (ii) the bio-interface optimization to improve resolution and tissue integration, (iii) the material biocompatibility and long-term aging.

Return electrodes in form of grids surrounding the stimulating electrodes could greatly increase the spatial contrast, and in specific cases also the spatial resolution, of electrical stimulation (Joucla and Yvert, 2009; Flores et al., 2016). A photovoltaic retinal implant with a ground grid has also shown the possibility to generate a high spatial resolution *in vivo* (Wang et al., 2012). 3D structures could further increase the spatial resolution with or without a ground grid by confining neurons in a stimulating area (Djilas et al., 2011) or bringing the electrode in the vicinity of the targeted neurons (Mathieson et al., 2012*)*. Using such 3D geometries, we have previously demonstrated that the remaining retinal tissue of blind rats can mold into 3D wells such that bipolar cells can be isolated in regular columns for a selective stimulation (Djilas et al., 2011). Others have proposed protuberant structures to induce a selective stimulation of retinal cells (Koo et al., 2006; Kim et al., 2008). In parallel mushroom shapes have been used in rigid Micro Electrode Arrays to improve the interaction between cells and the electronic device (Huys et al., 2008; Sasso et al., 2010; Fendyur and Spira, 2012).

We here investigated the possibility to combine 3D wells with the presence of protuberant mushroom electrodes on a flexible retinal implant. This study aimed at modeling the resulting current densities in the tissue and developing in parallel a simple microfabrication process compatible with the production of flexible implants having protuberant structures previously produced by others on rigid substrates such as silicon (Butterwick et al., 2009). Finally, using these prototypes, we investigated how the retinal tissue can interface properly with these new 3D complex structures with protuberant mushrooms in a well.

## Materials and Methods

### FEM Simulation

A finite element model (FEM) was developed using COMSOL^®^Multiphysics Version 5.2, Grenoble, France. The studied model consisted of a protuberant metallic electrode embedded in an insulating substrate and surrounded by a liquid environment with an electrical conductivity which represents the physiological environment, i.e., the retinal tissue. The interface between the insulating substrate and the liquid bio-environment is classically modeled using insulating boundaries described by Equation 1. For the electrode-electrolyte interface, Robin boundary condition described by Equation 2 is used as previously defined by Joucla et al. (2014). The conductivity values σ for the different materials were considered homogeneous and isotropic, and they are summarized in Table 1.

(1) ∇⁡V⁢n= 0

(2) $$\sigma \,\nabla V\,n=g\,\left(V_{m\,e\,t\,a\,l}-V\right)$$

**TABLE 1:** Parameters used for the simulation.

**Material**	**Conductivity σ (S/m)**	**Relative Permittivity**
Physiological liquid	1.47e^–2^	81
Insulating material	1.31e^–18^	3.4
Conductive material	45.6e^6^	–

**FIGURE 1:**
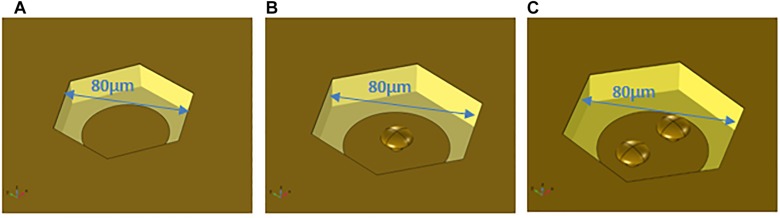
Different geometries modeled. **(A)** Cavity with a planar electrode in the bottom; **(B)** cavity with a planar electrode and one protuberance; **(C)** cavity with a planar electrode and two protuberances.

Three types of structures were modeled for comparison (Figure 1): a flat electrode in a cavity surrounded by a top ground plane, a single protuberant electrode in a cavity surrounded by a top ground plane, and a double protuberant structure embedded in the same cavity and with the same DC voltage being applied to the electrodes. For the three geometries the other parameters such as the cavity depth, width and length, the electrode diameter, or the pillar base diameter were constant, allowing the exact surface of the electrodes to be considered in each case.

### Microfabrication

**FIGURE 2:**
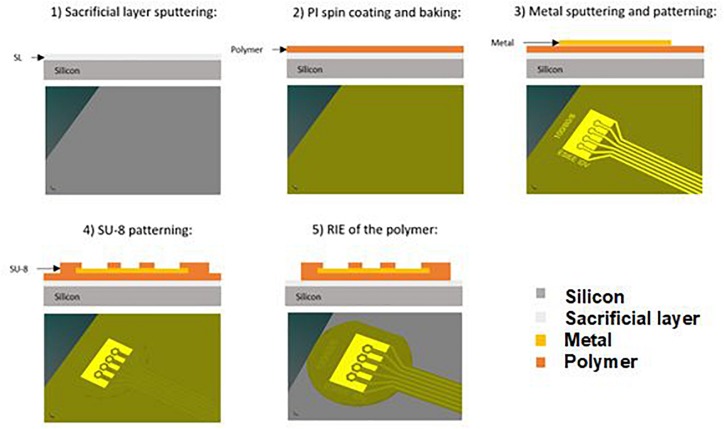
Fabrication process of the planar flexible implant.

The fabrication technology is based on the existing planar implant technology developed in our laboratory, to which new steps were added to introduce the protuberant electrodes into the 3D shaped wells. The fabrication process for the soft implants on a silicon wafer is summarized in Figure 2. (Step 1) A sacrificial layer composed of sputtered titanium (100 nm) and aluminum (500 nm) was deposited on a silicon wafer. (Step 2) 10 μm of polyimide (PI 2611 from HD MicroSystems) were spin coated and baked to create the substrate of the implant. (Step 3) A layer of titanium (100 nm) and gold (500 nm) was deposited by sputtering and patterned using photolithography to simultaneously define the electrodes, tracks and pads. (Step 4) SU8 2002 (from MicroChem) was spin-coated to form a 2 μm encapsulation layer to protect the metallic parts and, using a second photolithography step, openings were created on the pads and electrodes. (Step 5) Finally, 500 nm of aluminum were sputtered and patterned using an additional photolithography step followed by wet etching to create a mask to define the shape of the implant. The polymer was etched to pattern the shape of the implant by means of reactive ion etching (RIE), using a gas mixture of argon (Ar) and oxygen (O_2_) at 120 W and under a controlled flow rate. The aluminum layer acts as a stop layer for the polymer etching and it is chemically removed once the process is finished.

**FIGURE 3:**
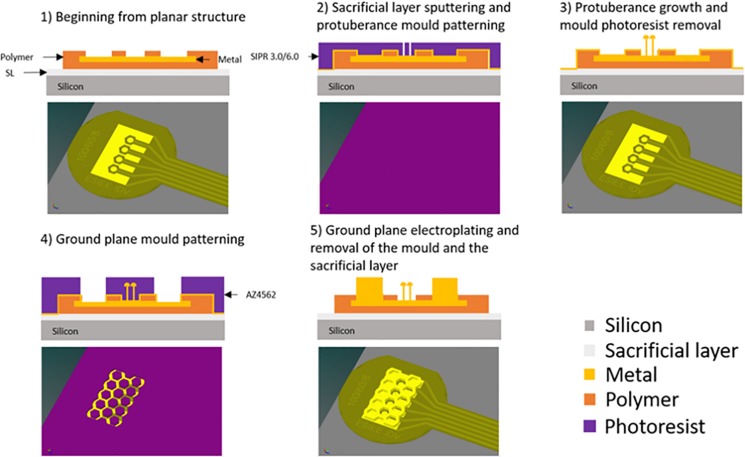
Fabrication process of the 3D structures on the flexible implant – Due to the small size of the holes compared to the implant, the software was unable to produce an image where such features can be appreciated while keeping the large view of the whole implant.

The fabrication process of the 3D structures on the flexible implant is summarized in Figure 3. Indeed the process continues after the patterning of the implant shape to produce the requested 3D structures (top ground grid and pillars at the bottom).

Initially, the 3D structures were fabricated in copper and then encapsulated in parylene to avoid any contact between the tissue and the copper. Copper electroplating is a well-known process in microfabrication technologies, which allowed us to fabricate the first prototypes fast and in a reliable way. Once the technology was established and consolidated, we were able to do *in vivo* experiments to assess its feasibility. Then, we transferred the technology to a biocompatible metal that does not need to be encapsulated i.e., gold.

(Step 1) On the whole wafer, we deposited a thin seed layer consisting of 50 nm titanium and 150 nm of either copper or gold, depending on the material used for the 3D structures. (Step 2) A photoresist (SIPR 3.0 or 6.0 from ShinEtsuMicroSi) was deposited and patterned to create the mold for protuberant electrodes. (Step 3) The wafer was placed in a copper or gold solution and a constant current (100 mA for copper and 0.1 mA for gold) was applied to electrodeposit the metal, thus creating the protuberances. The first photoresist was cleaned in solvent. (Step 4) A new thick photoresist (15 μm) layer was used to create the ground plane mold. (Step 5) Once the photolithography step was completed, the wafer was placed for a second time in the electrodeposition solution under the same growth conditions to obtain the ground plane. Then wafers were cleaned, and the initial seed layer etched. (Last step – not represented on Figure 3) A thin photoresist protection was applied before peeling off the implants to protect the electrodes and contacts from any corrosion. To peel off the implants from the silicon wafer, the aluminum layer below the implant was dissolved by electro erosion.

After cleaning, in the case of the copper devices only, an additional thin layer of parylene C (2 μm) was used to encapsulate the whole structure and ensure biocompatibility.

### *In vivo* Histology

The 3D structures have been implanted in sub retinal position in blind rats (P23H) to check the structural plasticity of the retina. Indeed, P23H rats (Machida et al., 2000) are considered as a reference model for retinis pigmentosa degeneration, since the rods’ degeneration is comparable to clinical cases observed on patients progressively losing their photoreceptors. The correct position of the implant is monitored by optical coherence tomography (OCT), immediately after surgery, and regularly every week.

The implant is explanted after 12 weeks *in vivo* and animal sacrifice and cell labeling is done for confocal analysis. The eyes are removed and placed in phosphate-buffered saline (PBS, 0.1 M, pH 7.4). The implanted area is isolated using a 3 mm biopsy punch. This fragment is fixed by incubation overnight at 4°C in paraformaldehyde in PBS (4% wt/vol) and then rinsed in PBS. For immunolabelling, retinal fragments are incubated in a blocking solution [10% bovine serum albumin (Sigma, St. Quentin Fallavier, France), 2% Triton X-100 (Sigma), 0.5% Tween 20 (Sigma) and 0.1 g/l Thimerosal (Sigma) in PBS] for 1 h at room temperature. They are then incubated for 3 days at 4°C (with slow stirring), followed by incubation at room temperature for 2 h with primary antibodies in blocking solution. The antibodies used are polyclonal antibodies directed against Chicken anti Glial Fibrillary Acidic Protein (1:100, LifeSpan Biosciences, Seattle, WA, United States), Rabbit anti Iba1 (1:500, Wako Sobioda, MONTBONNOT St. Martin, France) and a monoclonal antibody directed against mouse Goα (1:200, Merck-Millipore, Darmstadt, Allemagne). The fragments are rinsed and then incubated with secondary antibodies: goat anti-Chicken IgY Alexa 647, goat anti-rabbit IgG Alexa 488, and goat anti-mouse IgG Alexa 594 (1:500, Molecular Probes, Invitrogen, Eugene, Oregon) for 2 days at 4° followed by incubation at room temperature for 1 h. The implant/retina ensemble is rinsed and mounted, in permanent mounting medium (MM France, Brignais, France), on a microscope slide, for viewing under an upright confocal microscope from Olympus (FV1000 laser-scanning confocal microscope). 4′,6-diamidino-2-phenylindole (DAPI) counterstaining, AlexaFluor-488 and AlexaFluor-594 and AlexaFluor-647 can be detected by excitation with a 405 nm laser diode, a 488 nm argon ion laser, and 559 and 635 nm laser diode lines, respectively.

Thanks to this cell labeling technique, it is possible to count in which well we find bipolar cells compared to glial cells, and to proceed to a statistical analysis, typically to estimate the ratio of bipolar cells over total cell number, depending on the geometry and shape of the electrode. We need to keep in mind the limited number of *in vivo* experiments we can do. So we decided to focus on the largest cavities (80 μm) with more results available as small cavities are more difficult to fill.

All experiments were carried out in accordance with the recommendations of the European Community Council Directives (86/609/EEC) and with the ARVO (Association for Research in Vision and Ophthalmology) statement for the use of animals in ophthalmic and visual research. The protocol was approved by the French “Comité National de Réflexion Ethique sur l’Expérimentation Animale” under the reference #15258 2018052811521506 v1 in October 2018. The surgical procedure used to implant the prototypes has been described in detail elsewhere (Bendali et al., 2015).

### Statistical Analysis

Among the implants studied, some of the cavities could not be exploited because of difficulties to determine the number of remaining protuberances. Indeed, after surgery, some protuberances were broken (although we only implanted implants of good quality after fabrication, removing those with missing protuberances). For the determination of the influence of the cavity width, 16 cavities of 100 μm in diameter were exploited, 8 of 80 μm, and 3 of 60 μm. In the case of the influence of the number of protuberances, 12 cavities without protuberant structures were analyzed, 7 cavities with one protuberance, 9 with two protuberances, 9 with three protuberances, and 7 with four protuberances. The average number of total cells per implant is recorded using the explanted retina with previously described labeling technique.

### *In vivo* Physiology

As it is difficult to physically stabilize the 3D implants in the subretinal space, we demonstrate influence of the protuberant structures in acute conditions. Implantation is done just before the stimulation experiments and the retinal tissue does not have time to fully conform the 3D shapes compared to studies where the implant remained for several weeks. For this reason and in order to reduce the mismatch between the retinal tissue and the stimulating electrodes, a set of implants consisting of electrodes with protuberances but without cavities were fabricated for these tests. The idea is also to mimic in a short time a situation close to what is expected after long implantation when the retinal cells move into the wells. The employed device consists of four microelectrodes where one of them is planar, the second one has one protuberant structure, the third one has two protuberances and the fourth one has three three-dimensional structures. The size of the planar electrode is 60 μm and the height of the protuberances is around 7 μm.

The active site of the connected implant was acutely placed in the subretinal space of a 12 weeks old healthy Long Evans rat (Janvier Labs, France) while the flexible shank and base is placed on an adjacent platform. The healthy model enables a positive control of evoked potential via light stimulation to be compared with electrical stimulation. A craniotomy on the contralateral visual cortex is made to allow electrophysiological recordings. Initial anesthesia is provided through a 5% isofluorane induction for several minutes. Fixed anesthesia for the surgery and recording was done using intraperitoneal injection of ketamine 1000 (40 mg/kg, Axience, France) and domitor (0.14 mg/kg, Vétoquinol, France). Anesthesia is maintained with 1/3 initial dosage every 45 min. The animal is placed on a stereotaxic frame with body temperature maintained at 35°C. A sagittal incision is made from the ears to the eyes. Tissue is pushed aside to reveal the cranium. A craniotomy is achieved through drilling a window and removing the parietal bone and the dura mater. Gel foam soaked in cortex buffer is maintaining the integrity of the brain during the implantation.

The implant head was placed in the nasal hemisphere of the subretinal space contralateral to the craniotomy. Surgical microscope verified that the implant is placed in the subretinal position. Tropicamide (5%) was used for eye dilation while oxybuprocaïne provided a local anesthesia. A small sclerotomy was made on the dorsal sclera and a 1 mm incision gives access. Retinal detachment was achieved using basic saline solution as previously described (Roux et al., 2016). Immediately after the device implantation, the recording electrode array is descended into the monocular region of the primary visual cortex (V1M) contralateral to the implanted eye. Electrophysiolgy recording lasted about 3 h after the surgery and the animal was euthanized through intracardiac injection of pentobarbital (dolethal 1 mL).

Full field natural stimulation is provided by a white LED (Thorlabs, United States) having an intensity of 2.2 mW/cm^2^. Current controlled microelectrical stimulation is delivered using a stimulus generator (Multichannel Systems, Germany) with a stainless-steel needle as a counter electrode is placed subcutaneously on the lower back of the animal. Recording on the primary visual cortex is done using a 16-channel linear MEA (NeuroNexus) connected to a 16-channel amplifier (Multichannel System).

Intracortical recordings are taken while stimulation is delivered at the eye at a rate of 1 Hz and repeated 100 times to be averaged. Light stimulation lasted for 10–100 ms as usually used in brain light stimulation or optogenetics. Electrical stimulation is delivered to individual electrodes on the subretinal implant. Delivered electrical stimulation waveforms have a symmetrical charge balanced biphasic pulse of 1 ms at each phase and an interpulse interval of 1 ms. The 3 ms waveform of is repeated three times to match the duration of stimulations delivered by light and obtain a total of 10 ms stimulation. Variation of stimulus intensity, duration, cathodic vs. anodic, are compared to determine the response effect of the different geometrical configurations. Raw data has been sampled at 25 kHz and is analyzed for evoked potential. The signal is always bandpass filtered from 1 to 100 Hz and averaged over the 100 repetitions aligned by the trigger onset. The results compare the averaged response amplitude and latency of each acquisition.

## Results

### FEM Simulation

**FIGURE 4:**
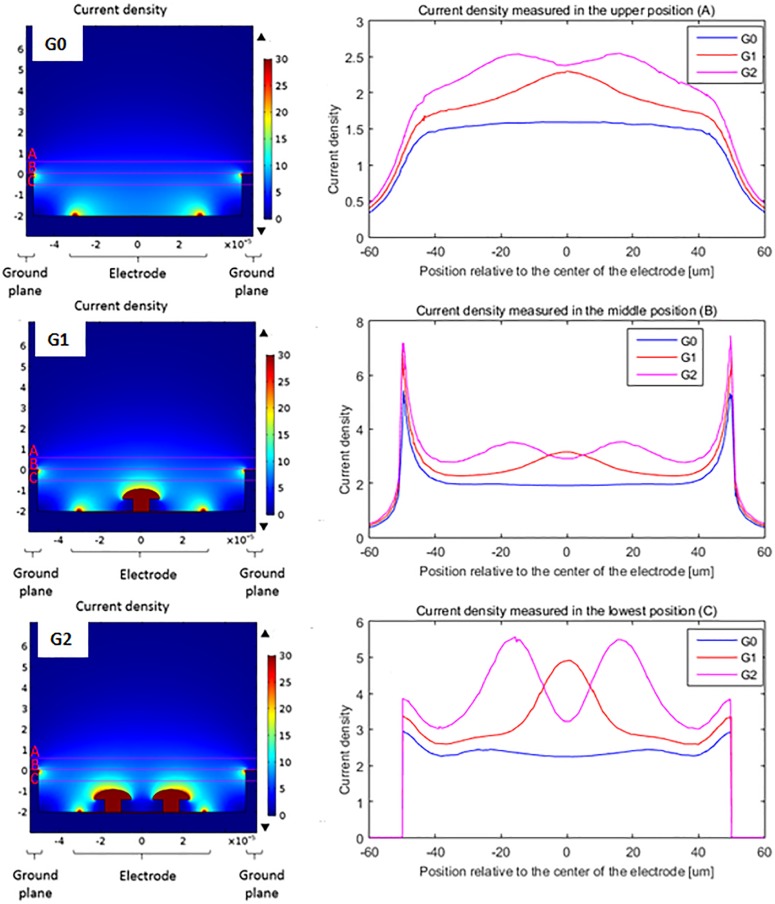
Comparison of the current density delivered by a flat electrode **(G0)**, a single protuberant **(G1)** and a double protuberant geometry **(G2)** in a two level configuration: ground plane and flat electrode plane are not at the same level – but same color scale (dark blue 0 to dark red 30 – units arbitrary here, as simulations done with a normalized applied voltage of 1V). Right side curves show the current density in A/m^2^ along the lines placed over the cavity (line A), at the ground plane level (line B) and inside the cavity (line C).

To assess the impact of introducing protuberant structures into a well on current distributions, Figure 4 illustrates the cross-sectional view of the current densities in a color code for the different conditions. The maximum and minimum values of current densities, corresponding respectively to upward and downward black triangles, depend on the computational meshing and so are slightly different, but the color scale is kept the same (dark blue – 0 to dark red – 30). These conditions have a ground plane on the superior part of the implant, a stimulation electrode centered at the bottom surface of the well for the planar geometry (G0) and protuberant mushrooms with an electrode on their surface (G1 and G2). In the planar geometry, a border effect is identified both at the border of the stimulating electrode in the well and the border of the ground plane: most of the current penetrates the liquid in this region of the electrode. In the protuberant geometries we appreciate a redistribution of the current density as higher current density is observed between the mushrooms and the ground plane.

Curve graphs show the comparison of the current density through a line over three different positions: over the cavity (line A), in the height of the ground plane (line B) and inside the cavity (line C). In the case of the measurement inside the cavity (line C), the protuberant structures change the distribution of the current density adding one (G1) or two (G2) peaks while the planar geometry (G0) shows two small peaks due to the edge effect of the planar electrode. Measurements done further from the electrode (line A and B) still show this effect but attenuated because of the distance. In the case of the line B measurements, the edge effect of the ground plane is also shown by the peaks in the edges, as line B corresponds to the top of the ground plane.

**FIGURE 5:**
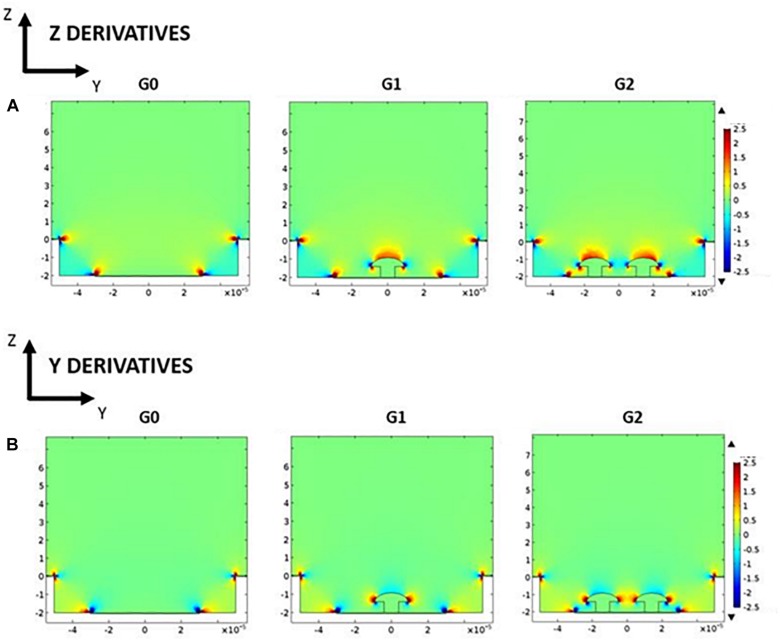
Potential derivatives along depth *Z* axis **(A)** and width *Y* axis **(B)** for the three studied geometries: G0, G1, and G2 (arbitrary color scale – from low value, blue, to high value, red).

In addition, Figure 5 shows the derivatives of the current density along the depth (*Z* axis) and the width of the well (*Y* axis) which is more representative of the current variations. We clearly see that the introduction of the mushroom pillars into the cavities concentrates the current variations inside the cavity where the bipolar cells are expected to be located. This spatial variation of the current density, according to the work activating function theory, should be responsible of neuron activation. Hence, neurons having moved into the well between the mushroom pillars and the ground plane would therefore be better activated.

### Microfabrication

**FIGURE 6:**
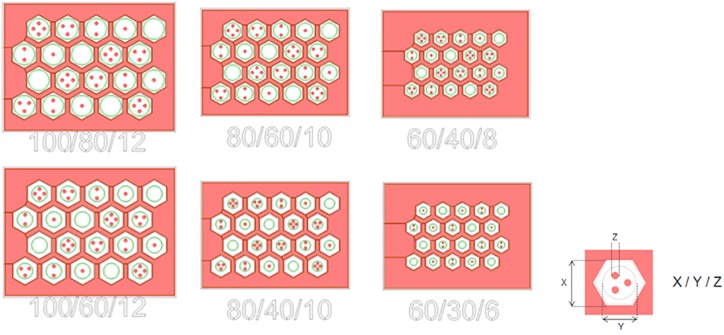
Mask of the six different configurations fabricated.

The size of the hexagonal cavities was varied from 100 to 60 μm and the diameter of the base of the protuberances from 12 to 6 μm. The number of protuberances was also varied from 0 to 4 in every cavity in order to study the adaptation of the tissue according to the number of protuberances. Figure 6 summarizes the different geometries resulting in the combination of different sizes of well and protuberances. Every implant has an identification code composed of one letter and three configuration numbers that represent the size of the different parts of the structure: X stands for well length, Y for planar electrode diameter and Z for protuberance base diameter. An additional letter was used to differentiate implants sharing a single configuration.

**FIGURE 7:**
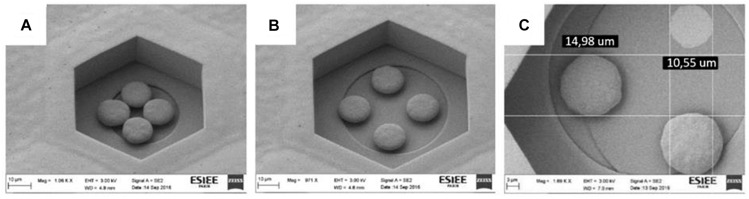
SEM picture of the four protuberant pillars made in copper in a 60/40/8 **(A)** – scale bar 10 μm – and a 80/60/10 **(B)** – scale bar 10 μm – structures. Detail of the diameter of a 3 protuberances structure with one of the protuberances detached in a 100/80/12 **(C)**, showing the base diameter of the pillar – scale bar 3 μm – For dimensions in **(C)**, 14.98 μm represents the diameter of the top of the mushroom while 10.55 μm represents the base of the pillar of similar mushroom.

Previous work in our group focused on the fabrication and *in vivo* characterization of cavities by means of silicon molding (Bendali et al., 2015). The main drawbacks of this technique are the time consumption required to create the silicon mold and the impossibility to obtain straight edges due to the isotropic (KOH) etching of the mold. Conversely, the electroplating method allows the control of the structure thickness by adjusting the time and the current in the bath during electroplating, and the well cavities can be defined with perpendicular walls at 90 degrees (Figure 7). In addition, the shapes have been defined to be compatible with other families of implants, like those based on photovoltaic effects (Mathieson et al., 2012; Wang et al., 2012).

**FIGURE 8:**
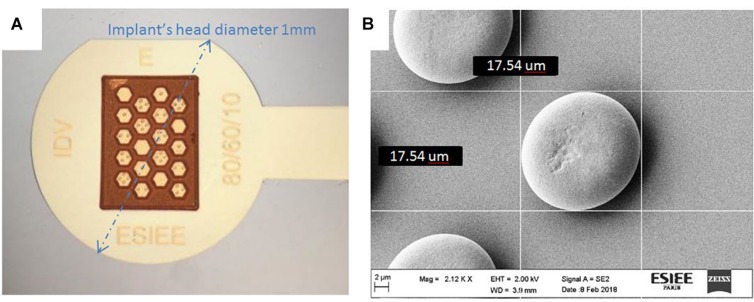
Device fabricated in gold: optical microscope image **(A)** SEM image of protuberances **(B)**; we observe small variations in the mushroom’s shape between Figure 7 and Figure 8, as electroplating parameters in copper and gold are different leading to different growth results.

As explained, devices were initially fabricated in copper due to its common use in microelectronics and its low cost compared to other techniques. However, copper is not a biocompatible material and it was covered with a layer of parylene (2 μm) to avoid any contact with the retinal tissue. Then a second rendition was fabricated using electroplated gold for the 3D structures. Growth conditions are different between copper and gold, leading to small changes in the mushroom’s shape. Optical (Figure 8A) and SEM (Figure 8B) images show the implant fabricated in gold (large view A with the implant’s head diameter being 1 mm and zoom on electrodes B with a base of 10 μm and the top of the mushroom of 17.5 μm).

### *In vivo* Histology

Previous studies in our group have already shown that retinal tissue is capable of adapting to and filling the cavities of a subretinal implant (Bendali et al., 2015). However, the cavities tested before were offering an angle of 54.7° due to KOH etching during mold creation, which may change the adhesion of the tissue compared to the vertical walls (90°) of the new devices described here.

**FIGURE 9:**
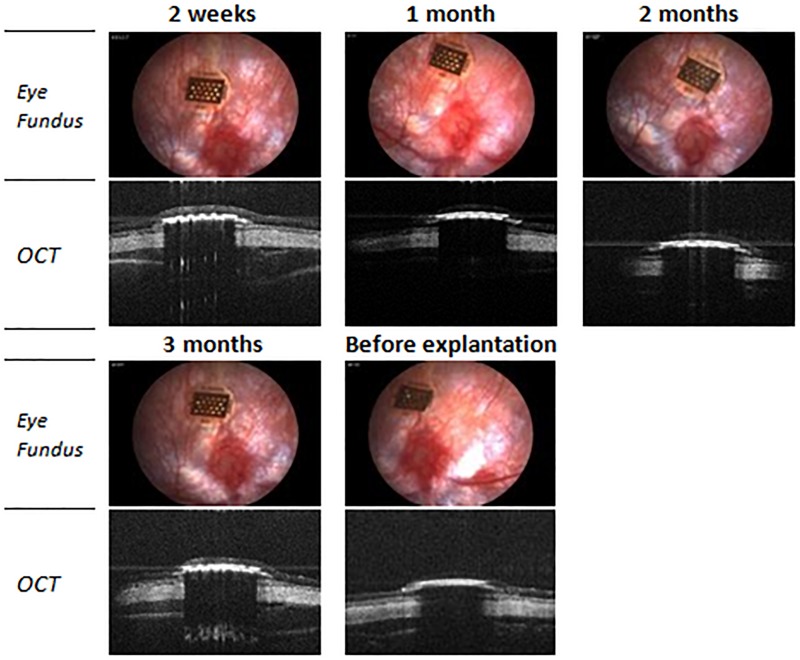
Optical coherence tomography (OCT) and eye fundus images of a 100/80/12 implant in subretinal position during the implantation period.

Optical coherence tomography (OCT) and eye fundus observation were used to verify the position of the implant during the 12 weeks of implantation (Figure 9). The results showed that the implant was stable in time over 3 months, did not cause retinal detachment, and did not damage the retina. In addition, OCT allowed us to visualize an absence of inflammation in presence of the implant under the retina.

Data from implants ranging from a well length of 100 to 60 μm (X dimension) and without protuberances inside were exploited. For comparison purposes, since the size of the cavities and the space occupied by the protuberances inside was different, the number of bipolar, glial and other types of cells has been counted for every cavity and then normalized regarding the total number of cells in the cavity.

**FIGURE 10:**
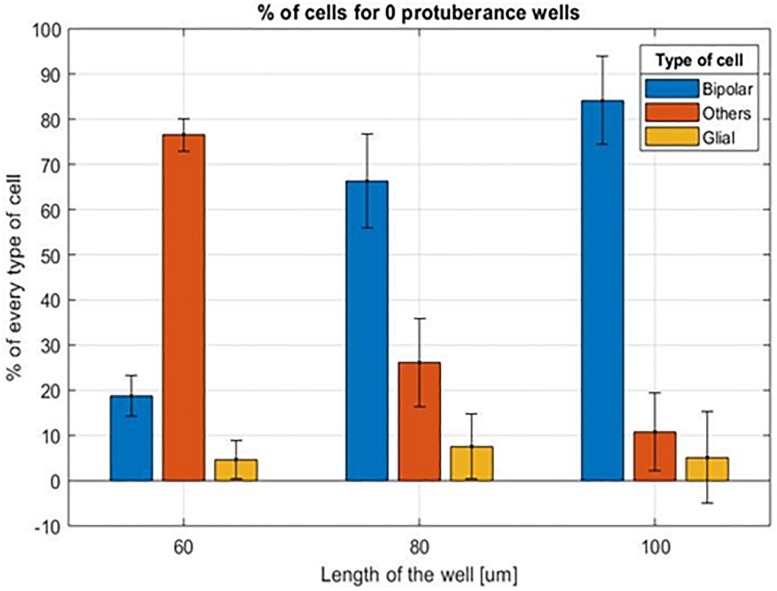
Mean percentage of cells for the different cavity sizes without protuberances inside.

The first relevant aspect is the influence of the diameter of the cavity on the molding of the retina onto the 3D structures. Figure 10 represents the percentage of cells of every type in the different cavities. The graphic shows a direct relation between the number of bipolar cells and the width of the cavity: 60 μm width cavities have an 18.75 ± 10.10% of bipolar cells which increases to 66.29 ± 8.67% for the 80 μm and to 84.10 ± 9.77% for the 100 μm ones. On the contrary, the number of other cells contained in the cavities is inversely proportional: for the 60 μm width cavities, there is a 76.59 ± 7.25% of other cells, 26.16 ± 9.75% for the 80 μm ones and 10.79 ± 10% for the 100 μm ones. We also observe that the number of glial cells almost remains constant for the three different cavity sizes: 4.66 ± 4.25% for the 60 μm, 7.55 ± 3.59% for the 80 μm and 5.11 ± 4.48% for the 100 μm.

**FIGURE 11:**
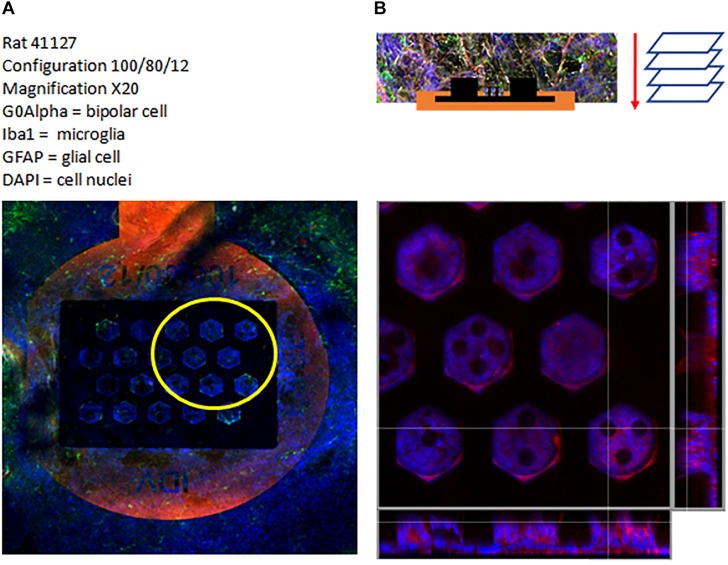
Confocal image of a 100/80/12 implant: general view of the implant **(A)**, top view and cross section view of the 3D region **(B)**. Labeling: G0Alpha (red), Iba1 (green), GFAP (brown) and Dapi (Blue).

This, again, demonstrates that the residual retina is structurally plastic enough to mold itself into the vertical 3D implant cavities for a cavity of minimal width above 80 μm in diameter, as confirmed with the confocal images (Figure 11).

**FIGURE 12:**
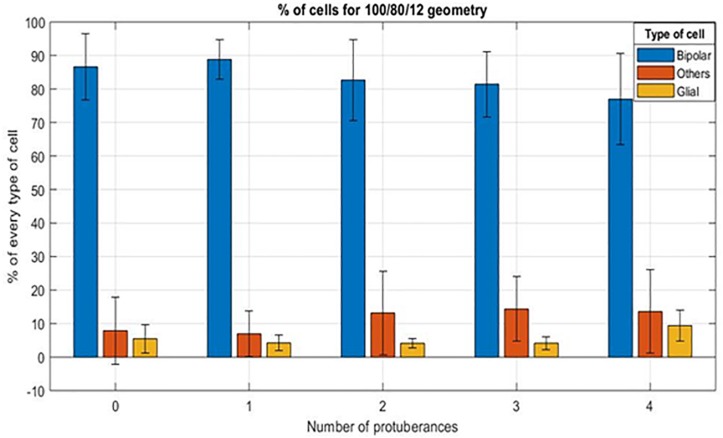
Mean percentage of cells for a 100/80/12 implant, depending on the number of protuberant structures (from 0 to 4).

The second aspect to study is the influence of the protuberances on the adaptation of the retinal tissue to the bottom of the cavity. For this purpose and considering the results of the influence of the cavity size, data from the 100 μm cavities are studied. Figure 12 shows the comparison of the mean percentage of every type of cells for the different structures contained in the 100/80/12 implants studied. Unlike in the case of cavity width, the number of cells of every type does not experience a significant variation for the different configurations. In the case of the bipolar cells the value for the cavities without protuberances is 86.64 ± 10.00% while for the opposite case of four protuberances it is 76.98 ± 13.63%. The average percentage of other cells also does not experience a significant variation being 7.88 ± 9.98% for the case of empty cavities, 13.18 ± 12.45% for the intermediate case of two protuberances and 13.62 ± 12.51% for the extreme case of four protuberances. As in the case of the different cavity width, the percentage of glial cells remains stable ranging from values of 4.13 ± 1.40% for the case of two protuberant structures to 5.47 ± 4.31% in the case of no protuberant structures, with the exception of the four protuberances cavities that has a value of 9.41 ± 4.65%.

### *In vivo* Physiology

**FIGURE 13:**
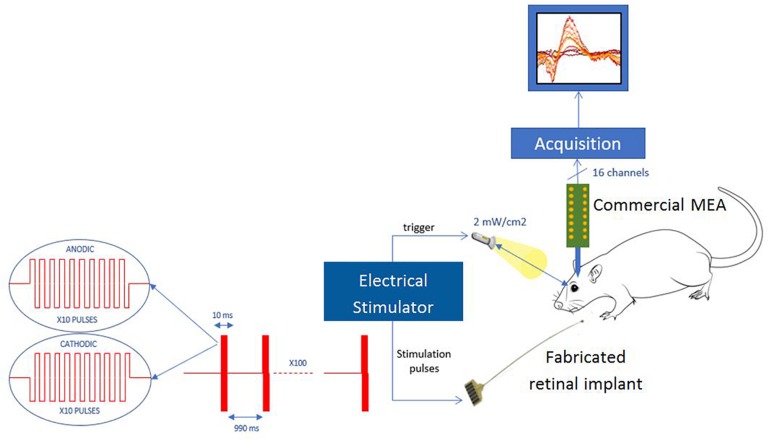
Measurement set-up for *in vivo* experiments.

**FIGURE 14:**
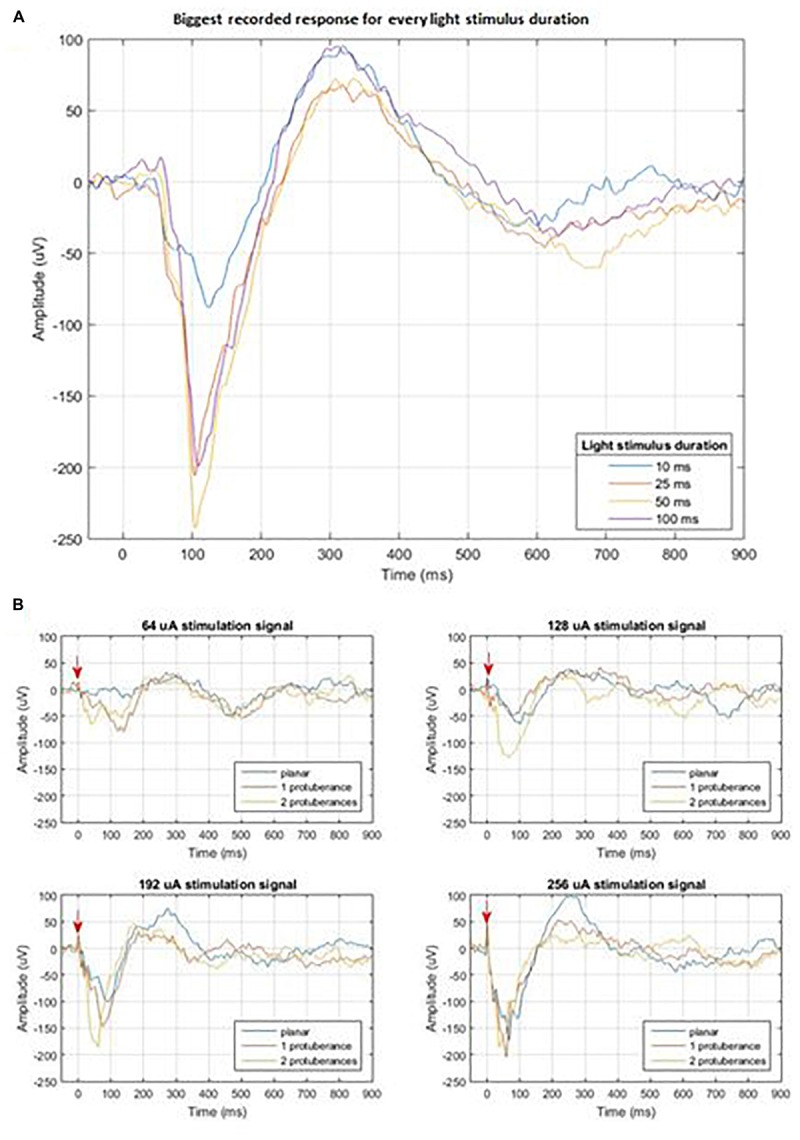
**(A)** Cortical measured response to light stimulus of different durations; **(B)** Cortical measured response of the three different stimulation electrodes for four different stimulation amplitudes: 64, 128, 192, and 256 μA. The red arrow indicates the moment of electrical stimulation.

Electrophysiological recording at the visual cortex of the brain allows for the observation of responses to light and electrical stimulation at the retinal. The general set-up is presented on Figure 13. Stimulation was delivered using pulses of 10 ms at 1 Hz with each recording lasting for 100 trials. Three stimulation electrodes were tested for comparison: classic planar electrode and two protuberant electrodes consisting of either one mushroom or two mushroom electrodes. Light stimulation delivered at the retina with different durations and a fixed intensity of 2.2 mW/cm^2^ was done to evaluate the cortical measured response. Figure 14A shows the cortical response to light stimuli at different duration measured by the recording electrode that had measured the largest response.

Electrical stimulation protocol consists of progressively increasing the current amplitude that is delivered at the different electrodes within the implant while recording at the same exact location in the visual cortex. Figure 14B shows the comparison among the measured responses to the stimulation of the three tested electrodes for the four highest current amplitudes: 64, 128, 192, and 256 μA. Our study did not show a difference in the local field potential response between stimulation waveforms that had started with cathodic or anodic first (data not shown). The plots in Figure 14B show that a threshold of current intensity must be reached in order to result an evoked potential similar to that elicited by light stimulation. This effect may be due to the smaller distance between the retina and the stimulating electrodes in the case of the protuberant ones and may be enhanced by the mismatch between the three-dimension geometries and the tissue.

## Discussion

In order to assess the influence of the microelectrode geometry on the current distribution, a finite element model of the microelectrode was developed, and three different geometries were modeled for comparison purposes.

Current density cross view and current density line graphs show that a high spatial variation of the current density is obtained by the introduction of protuberant structures on the top of the flat electrode. This spatial variation of the current density has been shown to have an influence on the neural activation. Hence, we expect that neurons having moved into the well between the mushrooms and the ground plane would therefore become highly activated. In addition, the use of cavities and a ground plane surrounding the stimulating microelectrode keeps the current in its vicinity, thus reducing the probability of activating other neurons.

Simulation results showed the interest of fabricating protuberant structures embedded into a cavity. To implant these structures in the retina and study their performance and the acceptance of the tissue to them, they should be preferably fabricated on a flexible polymeric substrate like polyimide or parylene for example. Two major techniques exist to create 3D structures: etching the silicon substrate or growing the structures by electroplating means. The electroplating method was chosen for its compatibility with soft implantable substrates.

The use of a microelectronics standard technique as copper electroplating allowed a fast development of the microfabrication process and the possibility of assessing the compatibility of these structures with the retinal tissue. Histological results showed that there is a direct relation between the size of the cavity and the number of bipolar cells that can enter in the cavity: cavities smaller than 80 μm are not suitable. Possible limitations of these results may come from the parylene used to encapsulate the metallic protuberant structures that might play a role due to its hydrophobic behavior.

Moreover, we tried to evaluate the influence of the number of protuberances for the same cavity size. For this purpose, 20 cavities per implant were defined, equally divided in five groups: cavities without protuberant structures, with one, with two, three, and four protuberant structures. All cavities were distributed in a manner to keep a constant separation of 16 μm among them. In order to maximize the number of electrodes per implant, this distance should be reduced, but a tradeoff is required to avoid sharp edges that could damage the retinal tissue. Therefore the results obtained in this first study can be exploited in a future study to optimize this parameter.

Another aspect that could have influenced is the distance between the cavities, which could prevent bipolar cells to descent into the cavities. Additional experiments would be needed to verify this hypothesis. These results also showed that the number of protuberances per cavity does not have a big influence on the rate of bipolar cells descending to the cavity.

Finally, after adapting the fabrication technique to use gold as microelectrode material, a preliminary *in vivo* stimulation experiment with these structures was conducted. Due to the surgical limitation of chronically fastening the flexible shank and base of the implant to the animal while maintaining the active site in the retina, an acute approach was taken. The results of this experiment showed that the implant is functional, and that electrodes with both protuberant and planar surfaces are capable of stimulating retinal tissue and evoking a response in the visual cortex. Recorded signals show that a smaller current is needed to generate a response when using protuberant electrodes. However, these results cannot be considered fully conclusive since the *in vivo* experiment was performed in only one animal and factors such as the position of the implant may play an important role.

Additional experiments must be conducted in the future to corroborate these preliminary results. Furthermore, in order to evaluate the 3D structures originally designed, a chronic implantation of the connected device must be achieved in order to ensure a reattachment of the retina to the device and that cells of the retina can occupy the 3D surface as in the biocompatibility results.

## Conclusion

The work presented in this article intends to address one of the problems of the retinal electrical stimulation devices: the specificity of the stimulation. For this, we propose the use of a novel microelectrode geometry that includes three main elements: cavities, protuberant microelectrode structures and a ground plane around the microelectrode structures.

The finite element simulation of the proposed structures showed that a redistribution of the current density is obtained around the protuberances. This redistribution of the current density may increase the probability of activating the neurons in its vicinity.

Considering the results of the simulation and their interest for retinal stimulation, a microfabrication process was developed to create these microelectrode structures on flexible substrates to create implantable devices. These devices were implanted on animals to perform a histological study and assess the feasibility of using these structures in a real retina. Results showed that a minimum width of 80 μm must be used for the cavities and that the number of protuberances per cavity does not have an important influence on the rate of bipolar cells.

Finally, a preliminary *in vivo* electrical stimulation experiment was performed. Considering that this experiment was performed only in one animal and it is not statistically significant, the results showed that the device is able to stimulate the retina.

In order to determine the performance of the presented microelectrode structures, more *in vivo* stimulation experiments should be conducted in the close future.

## Ethics Statement

All experiments were carried out in accordance with the European Community Council Directives (86/609/EEC) and with the ARVO (Association for Research in Vision and Ophthalmology) statement for the use of animals in ophthalmic and visual research.

## Author Contributions

PL developed the modeling part in collaboration with MG, and fabricated the devices in collaboration with LR. GL supervised and coordinated the first part of the research. MV, JD, and ED were in charge of the implantation procedures and image analysis. DN performed the *in vivo* stimulation experiments in collaboration with PL. SP supervised and coordinated the second part of the research.

## Conflict of Interest Statement

The authors declare that the research was conducted in the absence of any commercial or financial relationships that could be construed as a potential conflict of interest.
